# Big Data-Enabled Solutions Framework to Overcoming the Barriers to Circular Economy Initiatives in Healthcare Sector

**DOI:** 10.3390/ijerph18147513

**Published:** 2021-07-14

**Authors:** Yiğit Kazançoğlu, Muhittin Sağnak, Çisem Lafcı, Sunil Luthra, Anil Kumar, Caner Taçoğlu

**Affiliations:** 1 Department of International Logistics Management, Faculty of Business, Yaşar University, İzmir 35100, Turkey; yigit.kazancoglu@yasar.edu.tr (Y.K.); cisem.lafci@gmail.com (Ç.L.); 2 Department of Information Management, İzmir Katip Celebi University, İzmir 35620, Turkey; muhittin.sagnak@ikcu.edu.tr; 3 Department of Mechanical Engineering, Ch. Ranbir Singh State Institute of Engineering and Technology, Jhajjar-124103, India; sunilluthra1977@gmail.com; 4 Guildhall School of Business and Law, London Metropolitan University, London N7 8DB, UK; a.kumar@londonmet.ac.uk; 5 Department of Industrial Engineering, Faculty of Engineering, İzmir University of Economics, İzmir 35330, Turkey

**Keywords:** circular economy, sustainable development, healthcare sector, big data tools, barriers, fuzzy best-worst, fuzzy VIKOR, waste issues

## Abstract

Ever-changing conditions and emerging new challenges affect the ability of the healthcare sector to survive with the current system, and to maintain its processes effectively. In the healthcare sector, the conservation of the natural resources is being obstructed by insufficient infrastructure for managing residual waste resulting from single-use medical materials, increased energy use, and its environmental burden. In this context, circularity and sustainability concepts have become essential in healthcare to meliorate the sector’s negative impacts on the environment. The main aim of this study is to identify the barriers related to circular economy (CE) in the healthcare sector, apply big data analytics in healthcare, and provide solutions to these barriers. The contribution of this research is the detailed examination of the current healthcare literature about CE adaptation, and a proposal for a big data-enabled solutions framework to barriers to circularity, using fuzzy best-worst Method (BWM) and fuzzy VIKOR. Based on the findings, managerial, policy, and theoretical implementations are recommended to support sustainable development initiatives in the healthcare sector.

## 1. Introduction

The healthcare sector is undergoing significant changes that constantly create new challenges, hindering the implementation of the current model, and negatively affecting it on various levels, especially in developing countries [1]. The usage of resources, materials, and energy has been increased dramatically in the healthcare sector over the years [2]. This increasing resource need and demand in the health sector has led to an increase in the usage of disposable medical equipment, and single-use medical supplies. A large number of these healthcare supplies are used once and then discarded [3], which causes huge disruptions and burdens from the environmental perspective. Most of these disposable medical types of equipment are plastic products that are frequently used for various medical applications. The non-biodegradable nature of these has a harmful effect on the environment [4]. As a result of these emerging trends, the healthcare sector itself has been affected, and seeks sustainable solutions.

Additionally, the effect of the COVID-19 pandemic has placed tremendous strains on medical equipment movements resulting from supply chain disruptions, on aspects such as the reverse action of disposal and recycling practices to eliminate infectious medical wastes [5,6]. The growing amount of hazardous and contagious healthcare waste created as a result of advances in patient treatment has posed a significant threat to the entire planet [5]. The healthcare sector accounts for approximately 4.6 percent of global greenhouse-gas emissions [7]. From the viewpoint of healthcare, the volume of waste generated during these periods of outbreak crisis has provided various opportunities for the implementation of principles driven by the Circular Economy (CE), invovlving treatment of medical waste and the local procurement/recovery of essential raw materials [8,9].

The circular economy can be described as a restorative and regenerative approach that contrasts with a linear economy, which regards resources and energy as purely disposable [7]. One of the main principles of CE is to protect the value and quality of products and materials, and maintain their value in the economic system by extending the end-of-life cycle of the product [10,11]. As a result, while CE can help address shortages on the input side of the healthcare industry during the COVID-19 outbreak, the generation of hazardous waste inhibits CE adoption on the output side [9]. The reason for this was supply problems during the pandemic, such as lack of raw materials in the face-to-face transaction structure, and incompatibility between needs and available products, including sizes and quantities [8,12].

Similar to CE, monitoring generated waste requires the use of several networks of smart technology such as artificial intelligence (AI), big data, and the Internet of Things (IoT) [5,13]. Therefore, Industry 4.0 provides tremendous opportunities for CE, in which products are being remanufactured, reused, and recycled at end-of-life cycles [13]. Especially, ongoing advancements in the healthcare field, coupled with the rise of big data, have culminated in a rapid and vast expansion of medical data [14]. Big data analytics (BDA) can create evidence for better care delivery by allowing the extraction of valuable information from collected data patterns in research, clinical care environments, and operational settings. In this context, industrial and academic experts are becoming progressively more interested in the potential benefits of big data technology in healthcare [15].

The absence of suitable infrastructure, and the existence of barriers to achieve an efficient healthcare waste management system, particularly in emerging economies, have been cited as a significant challenge in addressing waste [5,16,17,18]. Additionally, the current literature on the healthcare sector shows a gap in terms of the connection between CE and I4.0 technologies, particularly big data analytics. Thus, big data analytics can be a promoter force in the overcoming of healthcare barriers due to the requirement for more expanded applications of I4.0 technologies. In this context, the following research questions (RQs) addressing the research gap are proposed:

RQ1: What are the barriers to CE and these indicators’ significance in the healthcare sector?

RQ2: Can big data analytics and its tools be applied to the healthcare sector to propose solutions in terms of circularity practices?

In order to address the aforementioned RQs, the current literature on the healthcare sector was scrutinized, and CE perspectives were adopted to manage sustainability-related barriers Next, big data analytics and its tools were used for proposing solutions for each barrier to be overcome. For this purpose, this study initially points out the barriers related to CE in the healthcare sector. Following this, big data analytics was adapted to the healthcare sector to provide solutions. In this context, the main contribution of this study is the detailed examination of the current healthcare literature about CE adaptation, and the proposal for big data-enabled framework that matches and ranks the solutions to circularity-oriented barriers using fuzzy best-worst method (BWM) and fuzzy VIKOR methods.

The paper is structured as follows. In Section 2, theoretical background about barriers to CE in healthcare sector and big data tools are presented and explained in detail. In Section 3, the methodology is presented. In Section 4, the case study is discussed. In Section 5, discussions, and managerial, policy, and theoretical implications emerging from the findings are presented. Finally, in Section 6, the concluding remarks are presented.

## 2. Theoretical Background

### 2.1. Samples Preparation

The below-mentioned barriers to CE adoption in the healthcare sector, derived from the relevant literature, were scrutinized. These barriers to CE, drawn from the literature, were grouped under various dimensions. These determined barriers were classified under eight sub-dimensions as economic and financial, policy, organizational, environmental, social, human resources, managerial, and operational and technology. Table 1 presents the barriers to circularity for healthcare sector.

Technologies required for CE principle implementations in healthcare can have beneficial effects on the environment, including maximizing efficiency in usage of energy, providing cost-efficient solutions, and the elimination of the natural resource waste. However, the adoption of these technologies, equipment, and solutions requires high-cost investments. Especially, with the impacts of the COVID-19, the healthcare sector has experienced a rising shortage of medical supplies, thus, the pandemic created a devastating financial burden. In addition to the pandemic, the healthcare sector faces challenges arising from a lack of financial resources and capabilities, hindering investment in green technology and solutions and for environmentally circular applications [23,59]. The reason underlying individual hospitals and health systems’ failure to give the required attention to these eco-friendly medical supplies is due to the challenges in identifying the economic benefits of environmental investments in the sector. One of the reasons for the lack of attention to these environmental investments is associated with healthcare’s miscellaneous portfolio, which includes products of both low and high economic value [3,10]; thus, financial gains of these investments are difficult to determine.

Governments may have some shortcomings in CE-oriented policy-making, regulation, implementation, and incentives for the healthcare sector on waste generation, and policies for the management and improvement of this waste. As a result of the constraints caused by the lack of government laws and policies, waste produced within the healthcare system endangers the environment and human health in many ways, as they are not inspected and recycled. In this context, a key barrier to the health sector’s transition to CE, a more sustainable option, is the lack of policies that enable health systems to adopt green-focused solutions and encourage this transition. To strengthen and accelerate the transition into a more circular and sustainable system for the healthcare sector, an important role will be played by emerging technologies. However, some views in the current literature suggest government policies, business models, and management decisions have the power not only to accelerate the implementation of suitable technologies, but also hinder it [16]. In this context, the lack of favorable policies for using technology and unfavorable government legislation is a key barrier to this transition in the health sector and requires immediate action.

Supply chain practices in a healthcare environment are deeply connected to organizational conditions, such as establishing partnerships, allocating responsibilities and authority, and coordinating and organizing interface processes [20,60]. Therefore, organizational barriers play a key role in establishing the organizational design, culture, value, and standards necessary for the adaptation of CE in the healthcare sector. Barriers towards CE are especially caused by deficiencies in healthcare systems’ organizational culture, such as poor organizational design, misconceptions about sustainability issues, inconsistent healthcare culture and values, and lack of established standards for CE activities.

The current healthcare system jeopardizes natural resources, causing irrevocable damages to the environment. Therefore, safe management and treatment of resultant medical waste and disposals is essential for eliminating contagion and meliorating the standards for public health. Although safe management of medical waste and disposal is a requirement for the prevention and control of health-related infections, necessary to strengthen trust in services, and reduce the cost of service provision [61], this issue continues to impede the transition to CE. Additionally, the lack of environmental management contributes to the generation of hazardous chemical and substances, such as carbon/greenhouse emission, one of the most widely studied barrier in the literature for emerging countries [62,63,64,65,66]. The healthcare supply chain accounts for the vast majority of the sector’s global greenhouse gas emissions. Moreover, the idea that single-use (disposable) medical devices and supplies are safer than reusable ones is one of the greatest drivers of the dominance of single-use devices in the healthcare supply chain [7].

Social barriers are also key challenges against CE implementation in the healthcare sector. For instance, lack of awareness regarding the concepts of sustainability and circularity issues has been determined as the factor that prevents organizations from adopting advanced environmental conscious practices [67,68,69]. Apart from the lack of awareness of circularity on organizational and managerial perspectives, public interest and reaction are also substantial influences on adoption of circularity. Therefore, lack of public interest and reaction to circular equipment (reusable medical devices) hold organizations back, and prevent organizations pursuing circular trends [67,68,70]. Therefore, lack of public perception and commitment to environmental issues is also a considerable barrier to accomplishing CE practices.

Limited Human Resources (HR) capacities also hinder the healthcare systems’ implementations of CE. In this context, the following limitations to CE practices are emphaisized: resistance to change, lack of knowledge, training, expertise, and training in organizations for sustainable and circular actions [20,28,71]. In this context, another substantial barrier is limited knowledge among healthcare workers and professionals of environmental problems, resulting from insufficient HR capabilities.

Managerial barriers, such as lack of top management support and commitment about circularity, proactive strategies for environmental burden, and conflict of interest among stakeholders, also play a vital role in CE adoption. These obstacles can affect the moral judgments of employees, who may face a mismatch between their personal values and their need to comply with corporate rules [20,28,72]. Even when laws exist, lack of law enforcement and government support lead to low adherence to sustainability [73,74].

Capacity building (e.g., resources, skills, knowledge, tools, equipment, etc.) as operational and technology barriers towards CE adoption, is among the most frequently mentioned barriers in the literature [24]. In addition, the size and complexity of circularity in healthcare systems are considered as another major barrier that inhibits the improvement of green and circular healthcare systems [22]. Furthermore, the absence of suitable infrastructure is another crucial barrier for safe management of the healthcare waste disposal systems [5,16,17,18], and also lack of recycling options for medical devices and equipment in terms of their reusability and end-of-life cycle, causing detrimental effects on the environment. On the technical side, limited IT infrastructure and technologies in medical informatics can be determined as a barrier, i.e., lack of or insufficient technologies, poor quality of designs, standards, and codes, and inefficient performance on circular practices [75,76,77,78,79].

### 2.2. Big Data Tools in Healthcare Applications

The healthcare system is comprised of broad and complicated medical devices, and equipment that has a long end-of-life cycle. However, these devices have residual value at the end of their lifespan, and also have hygienic and quality risks [3]; thus, recycling of such medical devices has become a controversial issue. The problem of socially, environmentally, and economically sustainable disposal of healthcare waste has become even more complex, especially with the highly infectious waste from COVID-19 patients and healthcare professionals [5]. If medical waste is not appropriately managed and disposed of, it poses significant health threats, such as potential infection and injuries to healthcare workers [80], and results in serious damage to the environment. The increase in healthcare waste is not solely due to demographic growth. Simultaneously, the increasing usage of disposable (single-use) materials resulting from the growing number and size of hospitals has contributed dramatically to the rise of these wastes [2,80]. The healthcare sector has detrimental effects on the environment; thus, healthcare professionals need to be aware of the environmental cost caused in order to reduce the materials used and energy consumed [2,81].

The CE concept is designed to be restorative or regenerative, as opposed to a linear economy, which absorbs limited resources, increases energy use [7], and generates waste. CE can be defined as an economic model that aims to reuse discarded or used products, resources, and materials as a production resource by providing protection and restoration to products and resources. It also deals with social, economic, and environmental issues at every stage of the system [3,82]. Therefore, the main objective of the CE is the elimination of waste by extending the product end-of-life cycle and closing the loop for the product and its components by recycling them back into the system [10]. The transition to a CE can provide efficiency measures by increasing cost savings, promoting more supportive environments and resilient cities, as well as improved and strengthened public health, healthcare delivery, and quality of life [83].

In the economic transition scenario for emerging economies in the healthcare sector, the adaptation and application of the CE in the I4.0 brings both opportunities and challenges [84]. Due to the inherent challenges and difficulties of healthcare data, designing and conducting big data technologies in this realm has opportunities and benefits [85]. Most healthcare systems are striving to replace outdated infrastructure and obsolete technologies with very scarce capital resources [86]. Hence, pervasive big data analytics infrastructures and computational technologies have begun to play a vital role in competitive and digital organizations, e.g., in the healthcare and pharmaceutical industry [87,88]. In recent years, big data has become essential in various fields, including public management, scientific research, business organizations, healthcare, manufacturing, social networking, and natural resource management [89].

Broad, diverse, and complicated computerized healthcare datasets that cannot be processed with traditional software, hardware, or data management tools are called big data in healthcare [25,90]. Big data refers to access, collects and store a large volume of data that is difficult to utilize using traditional data processing methods and platforms [89]. These modern big data technologies evaluate these vast amounts of data in a short time, and generate predictive models through machine learning, and statistical techniques [91]. In this context, big data analytics employs tools such as machine learning algorithms, and facilitates exploration of meaningful decisions through understanding the structure and relationships of data [92].

Machine learning tools can be described as a subset of artificial intelligence (AI), which incorporates algorithmic approaches, allowing computers to solve problems without the need for complex computer programming [93]. The integration of machine learning technologies into the healthcare sector and clinical practice has the potential to enhance healthcare quality [94]. The key areas of use of machine learning tools in the healthcare sector are clinical workflow design, training of healthcare workers, healthcare professional’s performance, forecasting of the potential problems and diseases, and legal and ethical application practices [93]. Machine learning techniques can be integrated with other soft computing techniques to improve results, depending on their performance and reliability [95]. Depending upon the increasing significance of quality benchmarks in public assessments and setting reimbursement rates, it might be valuable to train machine learning algorithms to direct consumers toward clinical activities that boost quality outcomes [94]. In addition, the use of machine learning in complex health systems can support the provision of infrastructure in the field of medical informatics by receiving information from patients through electronic records.

The main description of optimization generally refers to maximization or minimization of a numerical problem by providing a function, and finding the most desirable solutions to a problem. Optimization can be adapted into all engineering regulations, as well as other fields of data mining [96]. The usefulness of maintenance optimization algorithms differs significantly based on their capability to calculate an optimal solution in the least amount of time and money [97].

Artificial neural networks can be defined as a computational approach that focuses on mathematical structures and models that have a series of connected processing elements, configuration, and function that perform parallel. Artificial intelligence offers many technological advances to healthcare services, such as cost-effective and optimal healthcare services in real-time, efficient and reliable collaboration among interdisciplinary stakeholders, and solutions for non-traditional care environments, transforming the healthcare workplace and workforce, and introducing novel and different health information systems [86].

Cloud computing is generated from the development and integration of the many independent computing approaches and technologies that refer to utility computing, on-demand services, grid computing, and self-service internet infrastructures [98]. Cloud computing technologies encourage and reinforce IT capabilities so that they can be accessible at all times and from every location [99,100]. Cloud-based clinical big data analytics offer intelligence for more reliable and sustainable health tracking [101]. Cloud computing can provide various benefits to organizations such as the minimization of technology-related costs (capital, operational expense saving, and labor cost) and accordingly better healthcare services [102,103], improved infrastructure and, efficient usage of resources, and increased technology standards and expertise [100,104]. Additionally, there are serious obstacles in medical informatics (e.g., medical data collection, analysis, management, and proposing a solution) resulting from its expense-related issues [105,106]. Furthermore, cloud computing ensures scalability, flexibility, and productivity increase in IT infrastructure, eliminates costs of energy and power. Observance with legislation, development of healthcare services, enhanced operating processes and procedures, and accurate decision-making capability are some important examples of cloud in healthcare practices in terms of managerial, legal, and operational perspectives [100].

Data mining is the method of observing and revealing previously discovered knowledge, patterns, and trends in datasets, which is used to create meaningful data for generating predictive models [107]. Data mining technologies comprise a number of different approaches such as data summarization, clustering, finding dependency networks, classification, evaluating changes, and specifying outliers [108]. Usage of data mining can provide proactive solutions to the many barriers and challenges. These technologies aim to eliminate risks encountered by the organization, and facilitate decision-making processes of organizations through examining their patterns and trends [107]. Data mining can assist organizations in healthcare management, customer relationship management [109], detection of fraud, and abuses to determine unusual, abnormal, and outlier patterns [108].

Social network analysis can be characterized as a social structure that consists of a group of actors or networks of people who are connected by specific characteristics [110]. Thus, social network analysis usually deals with interactions and communications among individuals and groups [111]. Rather than the assessment of a single clinical discipline, social network analysis can be used in optimizing the entire system [112,113].

Statistical techniques are subsidiary mathematical structures that extract meaningful information and insights from the data by making predictions such as modeling, machine learning, data mining [114], multiple regression, discriminant analysis, logistic regression [115], nonparametric regression, and cluster analysis [86]. The application areas of statistical techniques can be categorized as accounting and finance, health and medicine, research, manufacturing, marketing, and business [115].

## 3. Methodology

In the methodology section of the study, the first step was setting the search strings for the identification and search for the barriers, and identifying publications related to our aim. For this purpose, search queries and strings were prepared before starting the search (see Table A1).

Based on a detailed investigation of the current literature review, the determined barriers to circularity in the healthcare industry and corresponding big data solutions to these barriers are presented. After identifying the barriers, these were categorized depending on their relevance or dimensions. Big data techniques were also determined to address each of these barriers. For the implementation step of the study, 30 experts were selected as decision-makers to decide the best-worst barriers in each dimension, and among all dimensions separately via interviews. Additionally, for related big data solutions, these experts were asked to apply VIKOR as a methodology. The best-worst method and VIKOR method were adopted in this study after a detailed investigation of the methodology sections of the studies. For instance, Gupta (2018) investigated a novel hybrid methodology consisting of the best-worst method and the VIKOR method for evaluating attribute weights, and subsequently rating the numerous alternatives (airlines) in the airline sector in terms of service quality [116]. Shojaei et al. (2018) proposed an airport evaluation and ranking model that combines the Taguchi Loss Function, the best-worst method (BWM), and the VIKOR approach [117]. Tian et al. (2018) investigated failure mode and effects analysis (FMEA) by combining a hybrid structure, fuzzy best-worst method, relative entropy, and a fuzzy VIKOR (VIsekriterijumska optimizacija I KOm-promisno Resenje) methodology to improve the performance of traditional FMEA [118]. After obtaining results from the respındents, the calculations and validation of the results must be performed.

Therefore, regarding these aforementioned barriers and relevant big data solutions discussed in the previous section of this study, the proposed barriers and big data solutions need to be validated through expert opinions. In this context, a group of seven experts, consisting of two professors from the faculty of medicine (25 and 20 years of experience, respectively), three industry experts, managing directors of well-known hospitals (14, 17, and 11 years of experience), and two governmental experts from the Ministry of Health (14 and 12 years of experience). They met to discuss the predetermined barriers and related big data solutions. Interviews were held with these industry experts and academics to discuss the proposed barriers to circularity in the healthcare industry. Although independent opinions of experts were based on the importance of specific barriers, we selected the most frequently mentioned barriers that play a major role in the adoption of CE initiatives in the healthcare sector.

**Figure 1:**
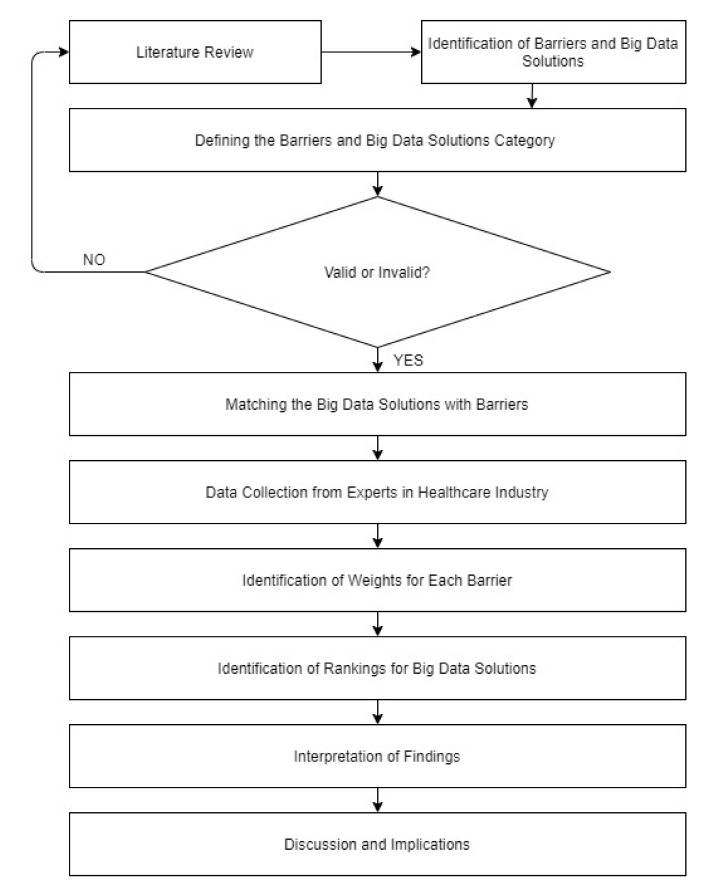
The overall flow of current research work.

After the validation process, the healthcare barriers to circularity were matched with Big Data solutions. Figure 1 displays the all-inclusive flow of current research work.

The fuzzy best-worst method was adopted to this study to calculate the relevant barrier and sub-barrier weights, and the fuzzy VIKOR method was used for ranking the big data analytics solutions for predetermined barriers.

The next section introduces the fuzzy set theory, the fuzzy best-worst method, and the fuzzy VIKOR method.

### 3.1. Fuzzy Set Theory

The process of decision-making involves some vagueness as a result of the ambiguity in the decision-making process. In attempt to deal with uncertainty, Zadeh [119] developed fuzzy set theory. This idea assists decision-makers in minimizing the subjectivity and ambiguity of human-related factors. A fuzzy set is referred to as a collection of objects with a range of grades.

### 3.2. Fuzzy Best-Worst Method

The best-worst method (BWM) is a comparatively new method, which was introduced by Rezaei [120]. As BWM is a vector-based method, it requires fewer pairwise comparisons compared to AHP or ANP, and the solution may be reached in a shorter time and with less complication. Furthermore, BWM comprises a mathematical model.

For weighing the criteria, BWM has five steps to follow.

Step 1: A criterion set, which is {$c_{1}$, $c_{2}$, …, $c_{n}$} is defined;

Step 2: The most important ($c_{B}$), and least important ($c_{W}$) criteria are identified;

Step 3: The most important criterion is compared with each of the other criteria. The best-to-others vector is identified by comparing the most important criterion with each of the other criteria. The best-to-others vector can be expressed as ${\tilde{A}}_{B}$ = (${\tilde{a}}_{B1}$, ${\tilde{a}}_{B2}$, …, ${\tilde{a}}_{Bn}$,). ${\tilde{a}}_{Bj}$ represents the fuzzy force of the most important criterion over criterion j. For example, ${\tilde{a}}_{BB}$ = (1, 1, 1);

Step 4: Each of these criterion needs to be compared with the least important criterion. The others-to-worst vector is identified by comparing each criterion with the least important one. The others-to-worst vector can be denoted as ${\tilde{A}}_{W}$ = ${\left({\tilde{a}}_{1W},\;{\tilde{a}}_{2W},\;\ldots ,\;{\tilde{a}}_{nW}\right)}^{T}$. ${\tilde{a}}_{iW}\;$rerepresents the criterion’s fuzzy force j over the least important criterion. For instance, ${\tilde{a}}_{WW}$ = (1, 1, 1)

Step 5: The optimal or ideal fuzzy weights ($\tilde{w_{1}^{*}}$, $\tilde{w_{2}^{*}}$, …, $\tilde{w_{n}^{*}}$) are calculated. The optimal fuzzy weights are ${\tilde{w}}_{B}$/${\tilde{w}}_{j}$=${\tilde{a}}_{Bj}$ and ${\tilde{w}}_{j}$/${\tilde{w}}_{W}$ = ${\tilde{a}}_{jW}$ for each pair. The maximum absolute differences are determined as $\left|\frac{\;{\tilde{w}}_{B}}{{\tilde{w}}_{j}}-{\tilde{a}}_{Bj}\right|$ and $\left|\frac{{\tilde{w}}_{j}}{{\tilde{w}}_{W}}-{\tilde{a}}_{jW}\right|$ for all j. All j values need to be formulated as a minimization model.$\;{\tilde{w}}_{B}$, ${\tilde{w}}_{W}$, and ${\tilde{w}}_{j}$ are triangular fuzzy numbers.

The mathematical model was developed as follows:

$$Minimize\;max\left\{\left|\frac{{\tilde{w}}_{B}}{{\tilde{w}}_{j}}-{\tilde{a}}_{Bj}\right|,\left|\frac{{\tilde{w}}_{j}}{w_{W}}-{\tilde{a}}_{jW}\right|\right\}$$

$$s.t.\left\{\begin{array}{c} \overset{n}{\underset{j=1}{\sum }}R\left({\tilde{w}}_{j}\right)=1\\ l_{j}^{w}\leq \;m_{j}^{w}\leq \;u_{j}^{w}\\ l_{j}^{w}\geq 0\\ j=1,\;2,\;\ldots ,\;n \end{array}\right.$$

$${\tilde{w}}_{B}=(l_{B}^{w},\;m_{B}^{w},\;u_{B}^{w}),\;{\tilde{w}}_{W}=\left(l_{W}^{w},\;m_{W}^{w},\;u_{W}^{w}\right),{\tilde{w}}_{j}=\left(l_{j}^{w},\;m_{j}^{w},\;u_{j}^{w}\right),$$

This mathematical model can be written as follows:

Minimize $\tilde{\xi }$

$$s.t.\left\{\begin{array}{c} \sum_{j=1}^{n}R\left({\tilde{w}}_{j}\right)=1\\ l_{j}^{w}\leq \;m_{j}^{w}\leq \;u_{j}^{w}\\ \left|\frac{{\tilde{w}}_{B}}{{\tilde{w}}_{j}}-{\tilde{a}}_{Bj}\right|\leq \;\tilde{\xi }\\ \left|\frac{{\tilde{w}}_{j}}{w_{W}}-{\tilde{a}}_{jW}\right|\leq \;\tilde{\xi }\\ l_{j}^{w}\geq 0\\ j=1,\;2,\;\ldots ,\;n \end{array}\right.$$

$$\tilde{\xi }=(l^{\xi },\;m^{\xi },\;u^{\xi }).$$

It can be supposed that ${\tilde{\xi }}^{*}=\left(\;k^{*},\;k^{*},\;k^{*}\;\right)$ and $k^{*}\leq \;l^{\xi }$ when $l^{\xi }\leq \;m^{\xi }\leq \;u^{\xi }$. The model can then be formulated as:

Minimize $\tilde{\xi }$

$$s.t.\left\{\begin{array}{c} \sum_{j=1}^{n}R\left({\tilde{w}}_{j}\right)=1\\ l_{j}^{w}\leq \;m_{j}^{w}\leq \;u_{j}^{w}\\ \left|\frac{l_{B}^{w},\;m_{B}^{w},\;u_{B}^{w}}{\left(l_{j}^{w},\;m_{j}^{w},\;u_{j}^{w}\right)}-\left(l_{Bj},m_{Bj},\;u_{Bj}\right)\right|\leq \;\left(\;k^{*},\;k^{*},\;k^{*}\;\right)\\ \left|\frac{\left(l_{j}^{w},\;m_{j}^{w},\;u_{j}^{w}\right)}{\left(l_{W}^{w},\;m_{W}^{w},\;u_{W}^{w}\right)}-\left(l_{jW},m_{jW},\;u_{jW}\right)\right|\leq \;\left(\;k^{*},\;k^{*},\;k^{*}\;\right)\\ l_{j}^{w}\geq 0\\ j=1,\;2,\;\ldots ,\;n \end{array}\right.$$

The optimal fuzzy weights, ($w_{1}^{*}$, $w_{2}^{*}$, …, $w_{n}^{*}$) can be obtained.

### 3.3. Fuzzy VIKOR

The VlseKriterijumska Optimizacija I Kompromisno Resenje (VIKOR) method was introduced by Opricovic [121], and Opricovic and Tzeng [122]. The main aim of the VIKOR method is identified as selecting the best alternative among others.

Opricovic [123] highlighted the steps of fuzzy VIKOR as the following:

Step 1: The fuzzy best and worst values are identified. The fuzzy best value ${\tilde{f}}_{j}^{*}=\left(l_{j}^{*},m_{j}^{*},r_{j}^{*}\right)$ and fuzzy worst value ${\tilde{f}}_{j}^{-}=\left(l_{j}^{-},m_{j}^{-},r_{j}^{-*}\right)$ are found as follows:

$${\tilde{f}}_{j}^{*}=\underset{i}{\max}\;{\tilde{x}}_{ij}$$

$${\tilde{f}}_{j}^{-}=\underset{i}{\min}\;{\tilde{x}}_{ij}$$

Step 2: The fuzzy difference (${\tilde{d}}_{ij}$) is found as follows:

$${\tilde{d}}_{ij}=\left({\tilde{f}}_{j}^{*}-{\tilde{x}}_{ij}\right)/\left(r_{j}^{*}-l_{j}^{-}\right)$$

Step 3: The separation values ${\tilde{S}}_{i}$, and ${\tilde{R}}_{i}$ of *i^th^* alternative are found as follows:

$${\tilde{S}}_{i}=\overset{m}{\underset{j=1}{\sum }}{\tilde{w}}_{j}\times {\tilde{d}}_{ij}$$

$${\tilde{R}}_{i}=\underset{j}{\max}\;{\tilde{w}}_{j}\times {\tilde{d}}_{ij}$$

where ${\tilde{S}}_{i}=\left(S_{i}^{l},\;S_{i}^{m},\;S_{i}^{r}\right)$ is a weighted score of the sum in terms of the separation value of *i*th option from $f_{j}^{*}$. In a similar way, ${\tilde{R}}_{i}=\left(R_{i}^{l},\;R_{i}^{m},\;R_{i}^{r}\right)$ determines the separation value of *i*th alternative from $f_{j}^{-}$. $w_{i}$ is the respective criterion’s weight, $C_{j}$;

Step 4: Q_i_ value is found: The value of ${\tilde{Q}}_{i}=\left(l_{i},m,r_{i}\right)$ is found by:

$${\tilde{Q}}_{i}=v\left[\left({\tilde{S}}_{i}-{\tilde{S}}^{*}\right)/\left(S^{-r}-S^{*l}\right)\right]+\left(1-v\right)\left[\frac{{\tilde{R}}_{i}-{\tilde{R}}^{*}}{R^{-r}-R^{*l}}\right]$$

where ${\tilde{S}}^{*}=min_{i}{\tilde{S}}_{i}$, ${\tilde{S}}^{-r}=max_{i}{\tilde{S}}_{i}^{r}$, ${\tilde{R}}^{*}=min_{i}{\tilde{R}}_{i}$, and ${\tilde{R}}^{-r}=min_{i}{\tilde{R}}_{i}$. v represents the weight for the maximum group utility, whereas $\left(1-v\right)$ shows the weight for individual regret. Then, ${\tilde{S}}_{i}$, ${\tilde{R}}_{i}$, and ${\tilde{Q}}_{i}$ values are defuzzified to reveal the crisp values. The crisp *S_i_*, *R_i_*, and *Q_i_* values are sorted escalatory order. The best alternative is identified through the corresponding minimum *Q_i_* value when the following two conditions are satisfied:

1. $Q\left(A^{\left(2\right)}\right)-Q\left(A^{\left(1\right)}\right)\geq DQ$, where $A^{\left(1\right)}$ and $A^{\left(2\right)}$ are the first- and second-best alternatives, respectively;
2. $A^{\left(1\right)}$ must also be found as the best alternative respecting *S_i_*, and *R_i_* values [124].

## 4. Case Study

This study mainly addresses the implementation phase, conducted in five chain hospitals located in Izmir, Turkey. However, it is important to note that these selected hospitals were chain hospitals, with branches operating in various regions of Turkey. Thus, the results of the study may be generalizable beyond the Izmir region.The major purpose is to determine the barriers of circular economy for the healthcare industry, and propose big data solutions to overcome each barrier.

The pairwise comparisons are conducted with 30 experts with different backgrounds. Table 2 displayed participant’s information during the study.

The proposed framework can be used for generic purposes where circular economy barriers in healthcare industry are studied; however, the findings are unique and cannot be generalized.

Table 3 demonstrates the best and worst criteria for each main criterion.

The weights of the main barriers were found as the step-by-step application of fuzzy BWM, and can be shown in Table 4.

According to the results in Table 4, economic and financial barriers were classified as the most substantial barrier to circularity with 0.275 weight. Policy barriers, operational barriers, and environmental barriers were also found as the other substantial barriers.

The weights of the sub-barriers within their own cluster, and the overall individual weights can be shown in Table 5.

Accordingly, “High cost requirement for circular technologies and implementations” was found as the most important barrier, with a weight of 0.131. Other important barriers were found to be “Unfavorable government legislation and execution on circular healthcare”, “Lack of financial capabilities and resources on environmental investments”, “Lack of safe management of medical waste in healthcare”, “Lack of circular policies, incentives, and regulations in healthcare”, “Lack of the top management support and commitment about circularity”, “Complexity of circularity in healthcare systems”, and “Challenges in identifying the economic benefits of environmental investments”.

After the implementation of the best-morst method, the next step was to identify ways to overcome these barriers. In this context, the VIKOR method was applied in the study in order to determine which big data-enabled technologies and solutions would appropriate for a smoother CE transition in the healthcare sector. Therefore, to overcome the barriers to CE initiatives, the experts investigated related big data technologies, including statistical techniques, cloud computing, data mining, artificial neural network, optimization, machine learning, and social network analysis.

According to the VIKOR application, Table 6 presents the rankings of the Big Data solutions for overcoming the barriers connected with a CE in healthcare industry. The ranking for the barriers is calculated via fuzzy VIKOR method.

Accordingly, cloud computing was found as the most important solution of big data to overcome the barriers to CE in healthcare industry. Other important solutions were found to be artificial neural networks, optimization, and data mining.

## 5. Discussion of Findings

The results suggest that 8 of 31 barriers were found as important: namely “High cost requirement for circular technologies and implementations”, “Unfavorable government legislation and execution on circular healthcare”, “Lack of financial capabilities and resources on environmental investments”, “Lack of safe management of medical waste in healthcare”, “Lack of circular policies, incentives, and regulations in healthcare”, “Lack of the top management support and commitment about circularity”, “Complexity of circularity in healthcare systems”, and “Challenges in identifying the economic benefits of environmental investments”. These combined have a total nearly 58% of importance weight. Three were determined among economic and financial barriers, two were policy barriers, and one each was environmental, managerial, and operational and technology barriers. This is not a surprise, as financial constraints were considered the most important obstacles in healthcare sector. In addition, policy barriers were directly related to governmental and legal issues, which were externally determined by governmental institutions; therefore, it is natural to find policy barriers as important.

Cloud computing was found as the most important big data solution to overcome the barriers to CE in the healthcare industry. In addition to that artificial neural networks, optimization, and data mining were suggested as other important solutions that can be adopted for addressing CE barriers and overcoming them. This was expected, as cloud computing can provide subsequent benefits to organizations, such as the minimization of cost for technology investments (capital, operational expense saving, and labor cost) and, accordingly, better healthcare services [102,103], overcoming poor infrastructure, insufficient resources, and lack of expertise and technology [100,104].

### 5.1. Managerial Implications

According to the results of the study, it can be deduced that insufficient or non-existent infrastructure investments of hospitals result from the high-cost of adopting and implementing these circular technologies. This result shows that hospitals need to take a more proactive role in improving the healthcare infrastructure, as it allows for rapid adaptation to continuously changing environments, which improves response times during pandemics [125]. In order to propose a solution for healthcare organizations with insufficient resources to invest in circular technologies and build infrastructure, cloud technologies are appropriate due to their infrastructure and different platforms, allowing users to install and run their applications in virtual servers based on their specific requirements [104].

Additionally, the lack of know-how, training, and expertise barrier is a substantial obstacle to the effective implementation of the circular activities, due to the recruitment, training, expertise, and knowledge of the medical staff involved. Thus, these processes require digital capabilities, skills, and in-job training, which can be used to increase the medical staff’s capacities. The artificial neural network (ANN) model could be valuable in the recruitment processes to determine and predict the staff’s expertise and performance-related information, and applied to in-job training to increase the capability and capacity of the workers. In addition, the results show that stakeholder management should be improved, as the healthcare industry’s multi-stakeholder structure needs to be digitally managed. The inimitableness of the healthcare sector, which has a substantial effect on the design of SCs, derives from its features such as the complexity of circular technologies, and the multiple tiers of stakeholders in the healthcare supply chain [60]. In this context, it is necessary to examine entire health processes and tiers in detail to reveal all the complexities of the healthcare supply chain for this transition. Process reengineering, data mining, and machine learning can be adapted to reveal the complexities of this barrier.

### 5.2. Policy Implications

Policy barriers are one of the two most important barriers, according to the global weights in all dimensions. Thus, policy implications have a causal relationship with the other barriers, and solutions to these barriers might provide effective solutions to the others. For instance, the underlying reason for most barriers is the lack of education and sufficient incentives. It is therefore vitally important to provide government legislation for education about circular practices. The competence to use digital technologies effectively needs to be a key focus in the healthcare sector. Students’ digital skills, analytical thinking, and decision-making skills are becoming incrementally important, and are highly related with the rapid transition and adaptability to the ever-changing conditions. This solution involves incorporating artificial intelligence and data mining in digital skills education. Furthermore, the optimization tool of big data can be beneficial in analytical thinking. Finally, optimization and statistical techniques can be adapted to the purpose of decision-making processes. Additionally, focusing on education can play a key role in reducing the deficiencies in know-how, training, and expertise. Government incentives are another crucial element due to the high cost of in-job training required for the development of workers. These cost-related investments must be encouraged and supported by the government. Data mining and machine learning tools can be used to reduce the financial burden of these requirements.

### 5.3. Theoretical Implications

The systems approach can be suitable for the healthcare sector, which is composed of multiple tiers and stakeholders. A systems approach can be described as a way of thinking in terms of connectedness, interactions, and context towards sustainability implementations, which can be a more valuable viewpoint than other theoretical approaches. A systems methodology entails gathering together experts from the appropriate disciplines into a team, which then uses a systematic framework to deliver a system, operating from needs to specifications and concept to implementation [126].

## 6. Conclusions

In the healthcare sector, the conservation of the natural resources is hindered by insufficient infrastructure for managing residual waste resulting from the usage of single-use medical materials, and by the environmental burden of excessive energy use. In addition, the COVID-19 pandemic has increased the challenges to the already strained healthcare sector. Additionally, economic crisis in the healthcare sector has had a huge impact, urging a faster transition to environmental sustainability, and the adoption of business strategies to eliminate residual waste and lower costs [127]. In this context, the circularity and sustainability concepts have become a must for healthcare to meliorate the sector’s negative impacts on the environment. Therefore, this study aims to identify the circular-related barriers in the healthcare sector and propose solutions.

In order to propose solutions to challenges, a detailed examination of the existing literature on healthcare was conducted, and it was understood that big data technologies and tools should be applied to the healthcare supply chain. Additionally, it was found that the connection between circularity (CE) and big data was a gap in the current literature hindering circularity practices in healthcare. Therefore, this problem requires special attention and solution suggestions. Usage of big data tools can provide social, economic, and environmental opportunities, and innovative solutions for circularity practices in the healthcare sector, especially during a pandemic, in which medical waste are ever increasing. In the health sector, waste management and recycling activities are key in reducing costs and, thus, enabling better patient care [61]. In this context, the main contribution of this study is the proposal for a big data-enabled framework for CE adoption in healthcare.

A detailed examination of the existing literature in the healthcare sector allowed the identification of barriers to CE transition. As a result, determined 31 sub-barriers were categorized under eight main dimensions. Fuzzy set theory was also incorporated into the study in order to deal with subjective and vague human decisions. For obtaining ranking and weights to these barriers, the fuzzy best-worst method was applied to find the best and worst main and sub-barriers. After this step, the fuzzy VIKOR method was used to rank the alternatives.

Accordingly, “High cost requirement for circular technologies and implementations” was found as the most important barrier. Other important barriers were “Unfavorable government legislation and execution on circular healthcare”, “Lack of financial capabilities and resources on environmental investments”, “Lack of safe management of medical waste in healthcare”, “Lack of circular policies, incentives, and regulations in healthcare”, “Lack of the top management support and commitment about circularity”, “Complexity of circularity in healthcare systems”, and “Challenges in identifying the economic benefits of environmental investments”. Cloud computing was found as the most important big data solution to overcome the barriers to CE in healthcare industry, and other important solutions were artificial neural networks, optimization, and data mining.

This study addresses a case study implementation in Turkey, which is a developing economy, thus it can be identified as a limitation of this study because of the generalizability of the results. When the implementation is applied to a developed country, different results may emerge. Another limitation is that the data used includes subjective judgments.

Future research might focus on the implementation of this proposed framework in other developing and emerging economies. Additionally, to better understand the implications of the framework, further studies could focus on determining the cause and effect relationship among barriers to CE and analytics of big data.

## Figures and Tables

**Table 1:** Barriers to CE in Healthcare Sector.

Main Dimensions	Code	Barriers	References
Economic and Financial Barriers	B1	High cost requirement for circular technologies and implementations	[2,13,19,20,21,22,23]
	B2	Lack of financial capabilities and resources on environmental investments	[19,23,24,25,26]
	B3	Challenges in identifying the economic benefits of environmental investments	[23]
	B4	Lack of demand for eco-friendly medical supplies	[20,21]
Policy Barriers	B5	Lack of circular policies, incentives, and regulations in healthcare	[7,13,19,20,21,22,25,26,27,28]
	B6	Lack of favorable policies for using technology	[25]
	B7	Unfavorable government legislation and execution on circular healthcare	[4,19,25]
Organizational Barriers	B8	Poor organizational design for CE principles	[20,21]
	B9	Current institutional misconceptions about sustainability	[20,22]
	B10	Inconsistency of healthcare cultures and values with CE principles	[24,29,30,31,32,33,34]
	B11	Lack of established standards for CE activities in healthcare	[20,21]
	B12	Lack of organizational readiness for CE activities	[24,31,33,35,36,37,38]
Environmental Barriers	B13	Lack of safe management of medical waste in healthcare	[13,39]
	B14	Lack of environmental management	[7,27,40,41]
	B15	Single-use medical devices and supplies	[2,7,42]
Social Barriers	B16	Lack of public perception and commitment to environmental issues	[4,20,21,23]
	B17	Lack of awareness about circularity	[19,24,36,43,44,45,46]
	B18	Lack of public interest and reaction	[21]
Human Resources Barriers	B19	Resistance to change	[20,25,47,48]
	B20	Limited knowledge-base on environmental problems among employees for CE activities	[23,24,31,35,36,49,50]
	B21	Lack of know-how, training, and expertise training for CE activities	[19,20,21,25,26]
	B22	Limited HR capacity for CE activities	[24,31,33,35,36,37,38]
Managerial Barriers	B23	Lack of top management support and commitment about circularity	[19,20,21,23,25,26,30,31,34,35,36,45,48,49,51,52]
	B24	Proactive strategies for environmental burden	[23,27]
	B25	Conflict of interest among stakeholders	[20,48]
Operational and Technology Barriers	B26	Challenges in identifying the impacts of environmental practices to clinical processes	[23]
	B27	Capacity building	[24,29,30,31,44,46,49,52,53,54,55]
	B28	Complexity of circularity in healthcare systems	[22,47]
	B29	Poor infrastructure	[5,13,24,46,49,51,53,54,56,57]
	B30	Lack of recycling options in medical supplies	[4]
	B31	Limited IT infrastructure and technologies in medical informatics	[25,26,58]

**Table 2:** Information about Participants.

Experts	Position of the Participants	Years of Work Experiences (in Total)	Experts	Position of the Participants	Years of Work Experiences (in Total)
1	Hospital Manager	21	16	Sustainability Expert	11
2	Hospital Manager	18	17	Sustainability Expert	9
3	Hospital Manager	14	18	Sustainability Expert	10
4	Hospital Manager	17	19	Circularity Expert	8
5	Hospital Manager	18	20	Circularity Expert	9
6	Head Doctor	24	21	Information Technology Expert	15
7	Head Doctor	20	22	Information Technology Expert	13
8	Head Doctor	19	23	Information Technology Expert	9
9	Head Doctor	23	24	Information Technology Expert	14
10	Head Doctor	27	25	Information Technology Expert	18
11	Supply Chain Manager	13	26	Ministry of Health Personnel	11
12	Supply Chain Manager	15	27	Ministry of Health Personnel	12
13	Supply Chain Manager	9	28	Ministry of Health Personnel	9
14	Supply Chain Manager	15	29	Ministry of Health Personnel	14
15	Supply Chain Manager	14	30	Ministry of Health Personnel	7

**Table 3:** The best and worst criteria for each main criterion.

Main Criteria	Best Criteria	Worst Criteria
Economic and Financial	High cost requirement for circular technologies and implementations	Lack of demand for eco-friendly medical supplies
Policy	Unfavorable government legislation and execution on circular healthcare	Lack of favorable policies for using technology
Organizational	Lack of organizational readiness for CE activities	Current institutional misconceptions about sustainability
Environmental	Lack of safe management of medical waste in healthcare	Single-use medical devices and supplies
Social	Lack of awareness about circularity	Lack of public interest and reaction
Human Resources	Lack of know-how, training, and expertise training for CE activities	Resistance to change
Managerial	Lack of the top management support and commitment about circularity	Proactive strategies for environmental burden
Operational and Technology	Complexity of circularity in healthcare systems	Limited IT infrastructure and technologies in medical informatics

**Table 4:** The Weights of Main Barriers.

Main Barriers	Weights
Economic and Financial	0.275
Policy	0.203
Organizational	0.087
Environmental	0.111
Social	0.056
Human Resources	0.073
Managerial	0.079
Operational and Technology	0.117

**Table 5:** Sub-Barrier Weights.

Barriers	Weights of Barriers	Sub-Barriers	Weights of Sub-Barriers	Overall Individual Weights
Economic and Financial	0.275	High cost requirement for circular technologies and implementations	0.477	0.131
		Lack of financial capabilities and resources on environmental investments	0.252	0.069
		Challenges in identifying the economic benefits of environmental investments	0.166	0.045
		Lack of demand for eco-friendly medical supplies	0.106	0.029
Policy	0.203	Lack of circular policies, incentives, and regulations in healthcare	0.310	0.063
		Lack of favorable policies for using technology	0.150	0.030
		Unfavorable government legislation and execution on circular healthcare	0.540	0.110
Organizational	0.087	Poor organizational design for CE principles	0.153	0.013
		Current institutional misconceptions about sustainability	0.095	0.008
		Inconsistency of healthcare cultures and values with CE principles	0.230	0.020
		Lack of established standards for CE activities in healthcare	0.137	0.012
		Lack of organizational readiness for CE activities	0.385	0.033
Environmental	0.111	Lack of safe management of medical waste in healthcare	0.601	0.066
		Lack of environmental management	0.246	0.027
		Single-use medical devices and supplies	0.153	0.017
Social	0.056	Lack of public perception and commitment to environmental issues	0.192	0.011
		Lack of awareness about circularity	0.660	0.037
		Lack of public interest and reaction	0.147	0.008
Human Resources	0.073	Resistance to change	0.100	0.007
		Limited knowledge-base on environmental problems among employees for CE activities	0.205	0.015
		Lack of know-how, training, and expertise training for CE activities	0.540	0.039
		Limited HR capacity for CE activities	0.154	0.011
Managerial	0.079	Lack of the top management support and commitment about circularity	0.640	0.051
		Proactive strategies for environmental burden	0.167	0.013
		Conflict of interest among stakeholders	0.192	0.015
Operational and Technology	0.117	Challenges in identifying the impacts of environmental practices to clinical processes	0.133	0.016
		Capacity building	0.122	0.014
		Complexity of circularity in healthcare systems	0.415	0.049
		Poor infrastructure	0.178	0.021
		Lack of recycling options in medical supplies	0.094	0.011
		Limited IT infrastructure and technologies in medical informatics	0.058	0.007

**Table 6:** Rankings of Big Data Solutions.

Rankings	Solutions	Q_i_
1	Cloud Computing	0.065
2	Artificial Neural Network	0.313
3	Optimization	0.455
4	Data Mining	0.496
5	Machine Learning	0.551
6	Statistical Techniques	0.749
7	Social Network Analysis	0.882

## Data Availability

No new data was created or analyzed in this study. Data sharing is not applicable to this article.

## Appendix A

**Table A1:** The Search Strings for Examination of Barriers.

**Healthcare**	(“healthcare sector” OR “medical” OR “health management” OR “public health” OR “health”)
**AND**	
**Big Data Techniques**	(“machine learning tools” OR “cloud computing” OR “artificial neural network” OR “optimization” OR “data mining” OR “statistical techniques” OR “social network analysis” OR “A/B testing” OR “data fusion and data integration” OR “natural language processing” OR “statistics” OR “association rule learning” OR “sentiment analysis” OR “genetic algorithms” OR “classification tree analysis” OR “regression analysis” OR “predictive modeling” OR “feature engineering”)
**AND**	
**Circular Economy**	(“circular economy” OR “green economy” OR “closed-loop economy” OR “circularity” OR “sustainability” OR “single-use medical materials” OR “medical waste” OR “greenhouse-gas emissions” OR “recycle” OR “reuse” OR “refuse” OR “reduce” OR “refurbish” OR “remanufacture” OR “repair” OR “recovery” OR “repurpose”)
**AND**	
**Barriers**	(“barriers” OR “challenges” OR “obstacles”)
